# Role of Trace Elements for Oxidative Status and Quality of Human Sperm

**DOI:** 10.4274/balkanmedj.2016.0147

**Published:** 2017-08-04

**Authors:** Galina Nenkova, Lubomir Petrov, Albena Alexandrova

**Affiliations:** 1 Laboratory of Free Radical Processes, Institute of Neurobiology, Bulgarian Academy of Science, Sofia, Bulgaria; 2 Department of Physiology and Biochemistry, National Sports Academy, Sofia, Bulgaria

**Keywords:** Metal ions, oxidative status, sperm

## Abstract

**Background:**

Oxidative stress affects sperm quality negatively. To maintain the pro/antioxidant balance, some metal ions (e.g. copper, zink, iron, selenium), which are co-factors of the antioxidant enzymes, are essential. However, iron and copper could act as prooxidants inducing oxidative damage of spermatozoa.

**Aims:**

To reveal a possible correlation between the concentrations of some metal ions (iron, copper, zinc, and selenium) in human seminal plasma, oxidative stress, assessed by malondialdehyde and total glutathione levels, and semen quality, assessed by the parameters count, motility, and morphology.

**Study Design:**

Descriptive study.

**Methods:**

The semen analysis for volume, count, and motility was performed according to World Health Organization (2010) guidelines, using computer-assisted semen analysis. For the determination of spermatozoa morphology, a SpermBlue staining method was applied. Depending on their parameters, the sperm samples were categorized into normozoospermic, teratozoospermic, asthenoteratozoospermic, and oligoteratozoospermic. The seminal plasma content of iron, copper, zinc, and selenium was estimated by atomic absorption spectroscopy. The malondialdehyde and total glutathione levels were quantified spectrophotometrically.

**Results:**

In the groups with poor sperm quality, the levels of Fe were higher, whereas those of Zn and Se were significantly lower than in the normozoospermic group. In all groups with poor sperm quality, increased levels of malondialdehyde and decreased glutathione levels were detected as evidence of oxidative stress occurrence. All these differences are most pronounced in the asthenoteratozoospermic group where values differ nearly twice as much compared to the normozoospermic group. The Fe concentration correlated positively with the malondialdehyde (r=0.666, p=0.018), whereas it showed a negative correlation with the level of total glutathione (r=-0.689, p=0.013). The total glutathione level correlated positively with the sperm motility (r=0.589, p=0.044).

**Conclusion:**

The elevated levels of Fe and the reduced Se levels are associated with sperm damage. The changes in the concentrations of the trace elements in human seminal plasma may be related to sperm quality since they are involved in the maintenance of the pro-/antioxidative balance in ejaculate.

Currently, infertility is becoming an increasingly widespread problem globally. In developed countries, infertility affects 3.5% to 16.7% of couples, and in less developed ones, 6.9% to 9.3% (1). Male infertility is as common as female infertility. About 30% of the issues involved with infertility are due to the man and another 30% due to the woman, while 30% result from complications with both partners, and in a further 10% of cases, the cause is unknown. Male factor infertility is a complex disorder. Many studies indicate that oxidative stress is one of the factors that influence negatively the fertility potential of spermatozoa. Oxidative stress is defined as a disruption in the balance between reactive oxygen species (ROS) production and the ROS scavenger capacity of the antioxidant system. ROS are a product of oxidative metabolism and at physiological levels are required for normal sperm functions such as hyperactivation, capacitation, and acrosome reaction (2). However, excessive ROS can be toxic, because in exceeding the available antioxidants, these highly reactive species can react with nearby molecules and can cause DNA, protein, and lipid molecules peroxidative damage within the cell (2). In turn, oxidative alterations provoke sperm dysfunction such as a loss of motility and viability, and impairment of sperm-oocyte fusion (3). Therefore, it is essential that the antioxidant system effective in order to overcome these conditions. Seminal plasma is rich in antioxidants (4) such as vitamins C (5) and E (6), glutathione (GSH) (6), urate (5), etc. In addition, there are 3 main enzymes that carry sperm protection: catalase (CAT), superoxide dismutase (SOD), and glutathione peroxidase (GPx) (7). The metal ions copper and zinc are co-factors of SOD, iron is a co-factor of CAT, and selenium is a co-factor of GPx. Therefore, the availability of these metals in the organism plays a beneficial role, because they influence the activity of the corresponding enzymes. However, iron and copper could act as prooxidants, catalysing the formation of hydroxyl (OH) radicals from superoxide anion radicals (O_2_-) and hydrogen peroxide (H_2_O_2_) (7,8). Hydroxyl radicals are highly reactive with a very short half-life and an extremely limited diffusion capacity. In practice, they attack and oxidize the first molecule they meet. Today it is assumed that OH have the most damaging effect on cell structures. The most vulnerable to oxidative attack are plasma membranes, due to the high percentage of polyunsaturated fatty acids in their composition. It has been suggested that the peroxidation of membrane lipids is largely responsible for sperm dysfunction (8). 

This dual role of metal ions provokes our interest in investigating the concentration of cоpper, zinc, irоn, and selеnium in normal and pathological human semen and their impact on oxidative stress occurrence, evaluated by the levels of malondialdehyde (MDA) and GSH.

## MATERIALS AND METHODS

### Chemicals

All reagents and chemicаls used for biоchemical analyses [2-thiobarbituric acid (TBA), trichloroacetic acid (TCA), HCl, reduced GSH and oxidized glutathione (GSSG), 5,5′-Dithiobis(2-nitrobenzoic acid) (DTNB), GSH reductase] were obtained from Sigma-Aldrich Chemie GmbH (Darmstadt, Germany).

### Statement

This research was performed in accordance with the Declaration of Helsinki for Human Researches [World Medical Association Declaration of Helsinki - Ethical principles for medical research involving human subjects. 59^th^ WMA General Assembly, Seoul, Republic of Korea (2008)]. The approval of the Ethics Committee of the Research Board was obtained.

### Patients and semen collection

Semen samples were obtained from men aged between 24 and 38 years attending a fertility clinic. Ejaculates were collected in sterile containers via masturbation after a sexual abstinence of 2 to 7 days. All participants were previously informed in detail about the objectives, tasks, and conduct of the survey. A written consent for participation in the research was taken from all patients.

### Semen parameter analysis

After liquefaction of the semen samples for 30 minute at 37 °C a standard analysis was performed within 1 hour according to World Health Organization guidelines (9) using a computer-assisted sperm class analyzer (Microptic SL, Barcelona, Spain). The software settings were those recommended by the manufacturer for the analysis of human sperm. Semen volume (mL), count (10^6/mL), and motility (%) were included in spermiograms.

To determine the normal and respectively abnormal morphology (%) of spermatozoa a SpermBlue staining method (Microptic SL, Barcelona, Spain) was applied. Ten μL of the respective ejaculates were pipetted onto slides. The smear was allowed to dry at room temperature. After that, the sample was placed in a fixing solution for 10 minute, and then stained in SpermBlue for 12-15 min. The morphology of at least 200 spermatozoa was analysed using the sperm class analyzer (Microptic SL, Barcelona, Spain) and a Leica DM 1000 microscope. Sperm morphology was assessed using 100x objective (immersion oil lens). Kruger’s criteria, where morphology <14% is considered abnormal, were used for evaluating spermatozoa morphology (10). 

The samples were then categorized according to their seminal fluid analysis parameters into the following groups: normozoospermic, teratozoospermic (with normal morphology <14%), asthenoteratozoospermic (with motility <40% and normal morphology <14%), and oligoteratozoospermic (with sperm concentration <15 million/mL and normal morphology <14%).

After semen analysis, the ejaculates were centrifuged at 3500 rpm for 15 minute to separate the seminal plasma from spermatozoa. The obtained fractions were stored at -80 °C until biochemical and elemental analyses.

### Measurement of lipid peroxidation

Lipid peroxidation was assessed by the quantity of the TBA reacting substances (TBARs), formed in the preparations, according to the method of Hunter et al. (11). To the semen samples (0.5 mL) were added 0.3 mL of mixture, containing 2.8% TCA + 5 M HCl + 2% TBA in 50 mM NaOH (2:1:2 v/v), which were heated at 100 °C for 15 minutes for color development. The samples were cooled and centrifuged at 1000 x g for 10 minutes. Then the resulting supernatant was separated, and the absorbance was read at 532 nm and 600 nm against appropriate blanks. The 600 nm absorbance was subtracted from that of 532 nm, because the former was considered to be a non-specific baseline. Every assay was conducted in triplicate using a Jenway 6305 Single Beam UV/Visible spectrophotometer (Bibby Scientific Ltd., Stone, Staffordshire, UK). The values were expressed in nmols MDA/mL, using a mоlar extinction coefficient of 1.56x10^5 M^-1^cm^-1^.

### Determination of total glutathione level

The total GSH (tGSH) was measured spectrophotometrically by the method of Akerboom and Sies (12) using DTNB. This colorimetric assay is based on the reaction between GSH and DTNB where TNB (5-thio-2-nitrobenzoic acid) is formed. TNB exhibits maximum absorbance at 412 nm. The intensity of the absorbance is proportional to the GSH level in the sample. A standard curve, prepared with known amounts of GSH, was used for determining the sample’s GSH concentrations. Every assay was conducted in triplicate using a Jenway 6305 Single Beam UV/Visible spectrophotometer (Bibby Scientific Ltd., Stone, Staffordshire, UK). The values were expressed in μmol/L.

### Determination of Fe, Cu, Zn, and Se levels

The iron, copper, zinc, and selenium concentrations in sperm were estimated by atomic absorption spectrophotometry, using a Perkin Elmer Zeeman 5100PC (Perkin-Elmer GmbH, Rodgau, Germany) provided with an HGA 600 graphite furnace programmer. Seminal samples were diluted 1:50 with deionized water to determine the zinc concentration and were directly measured by flame atomic spectroscopy, whereas a graphite furnace atomic absorption spectroscopy technique was used to assess copper, iron, and selenium after 1:10 dilution with deionized water. For quality control, standard materials (Merck, Darmstadt, Germany) were used as references. 

### Statistical analysis

Values were presented as mean ± standard deviation. For establishing the differences between groups an analysis of variance (one-way ANOVA) was performed, and for conducting pairways comparisons the post hoc test (Tukey test) was applied. At values of p<0.05, the differences were considered to be significant. For data analysis the Windows computing program Statistical Package for the Social Sciences “SPSS 16” (SPSS Inc., Chicago, IL, USA) was used.

## RESULTS

The average values of the sperm specimen parameters from the four investigated groups are given in Table 1. A significant decrease in the semen count, motility, and percentage of normal morphology was observed in the three abnormal groups when compared to normozoospermics; there were no significant differences in seminal volume. 

Significantly elevated levels of MDA were detected in the abnormal groups, as in the teratozoospermic group the level was increased threefold and in the asthenoteratozoospermic group fourfold in comparison to the normozoospermic group. The detected level of tGSH was almost twice lower in the asthenoteratozoospermic than in the normozoospermic group (Table 2). There were no significant differences between the tGSH level of the teratozoospermic and oligoteratozoospermic group. 

The concentrations of the seminal trace elements are presented in Table 3. The concentration of Zn and Se were significantly lower in the three abnormal groups than in the control group. In regard to Fe concentrations, they were higher in the abnormal groups, but a significant difference was registered only between the asthenoteratozoospermic group and normozoospermic group (4.25±0.53 vs 2.70±0.19 mg/L). We did not find significant differences in the level of Cu between the tested groups. 

From the studied trace elements, only Fe demonstrated a significant positive correlation with the MDA level (r=0.666, p=0.018) and a negative significant correlation with the tGSH level (r=-0.689, p=0.013). The seminal tGSH level correlated positively with sperm motility (r=0.589, p=0.044). 

## DISCUSSION

Trace elements play an essential role in all aspects of human physiology, including reproduction. Metal ions, among their many other functions, play essential roles in about one-third of enzymes (13). In terms of the pro-/antioxidant balance in the organism, the metal ions copper, zinc, iron, and selenium are very important, acting as co-factors of the antioxidant enzymes Cu, Zn-SOD, CAT, and GPx, respectively. Thus their availability in the organism should play a beneficial role, because their presence determines the respective antioxidant enzyme activity. However, iron and copper could act as prooxidants, inducing oxidative damage of the cell compounds. On the one hand, iron is a c]o-factor of catalase, the enzyme that catalyses the decomposition of hydrogen peroxide. Many authors have recognized H_2_O_2_ as the most toxic oxidizing agent for human spermatozoa (3). Therefore the decomposition of H_2_O_2_ is essential for preventing its toxic effect. Khosrowbeygi et al. (14) found a significantly lower catalase activity in patients with asthenozoospermia, asthenoteratozoospermia, and oligoasthenoteratozoospermia than in normozoospermic males. They concluded that the decreased seminal plasma catalase activity could be associated with poor sperm quality. On the other hand, it is well known that trаnsition mеtal ions such as iron and copper can generate the highly reactive hydroxyl radicals in the presence of H_2_O_2_ and O_2_- (Haber-Weiss reaction) or H_2_O_2_ (Fenton reaction) (7). A high concentration of iron has been found in whole semen - about 500 μg% - and the concentration in seminal plasma was reported as 315 μg% (between 265 and 365 μg%) (15), both obtained from normal fertile men. In comparison, the total iron in blood plasma is about 100 μg% (16). Our findings correspond to the above-mentioned concentrations with a mean value of 270 μg% (2.7 mg/L) iron in the seminal plasma. We found an increase of iron levels in the asthenoteratozoospermic versus the normozoospermic group, but there was no significant correlation between iron concentration and either sperm motility, morphology, or concentration. Skandhan et al. (15) reported cases with a much higher iron concentration, accompanied by motility of less than 10%. Huang et al. (17) also detected higher levels of iron in asthenospermic men than in normal controls, which were accompanied by higher levels of MDA. The authors observed a negative correlation between the MDA concentration and sperm motility in the abnormal groups (r=-0.28, p<0.05), but with no association among trace elements and MDA in seminal plasma. The incubation of human sperm with Fe^2+^ led to a significant reduction of the sperm motility at 5 ppm and an increase of MDA levels (18). These results suggest that increased lipid peroxidation in response to elevated Fe^2+^ levels may inhibit sperm motility. Our findings showed a significant positive correlation between the iron concentration in the samples and the lipid peroxidation, as well as a negative correlation between iron concentration and the tGSH level. The high MDA level and low tGSH level could be one of the causes of decreased motility and increased percentage of abnormal spermatozoa. MDA is an end product of lipid peroxidation and serves as an important tool in the measurement of lipid peroxidation. It is well known that spermatozoa are more susceptible to lipid peroxidation than other cells. There are two reasons for this: 1) during the final stages of spermatogenesis they discard most of their cytoplasm, along with cytoplasmic defence enzymes like CAT and SOD, and 2) their plasma membrane is rich in polyunsaturated fatty acids. There is a lot of evidence that MDA concentration is negatively associated with sperm motility, concentration, and normal morphology, and exhibits an inverse relationship with sperm-oocyte fusion (19). GSH is considered to be a major antioxidant within the cells. It is a co-factor required for the activity of GPx, which directly binds to ROS via its free sulphydryl groups. The presence of GSH in the extracellular space has a protective effect on the sperm plasma membrane, since it can detoxify cytotoxic aldehydes generated during lipid peroxidation (20). Significantly higher GSH levels have been detected in normozoospermic men than in groups with sperm defects and a significant relationship between seminal GSH and sperm motility (20) has been suggested - observations similar to our results. In turn, GPx is a selenoprotein and in fact the importance of Se in mammals is due to its role as a prosthetic group in the catalytic sites of the enzyme. Although we did not find any correlations between Se content, sperm parameters, and oxidative status, many authors have demonstrated a negative effect of low levels of seminal Se on the number and motility of spermatozoa (20). It has been shown that both Se (21) and Zn (22) play an essential role in testicular growth processes, testosterone secretion, and spermatogenesis. Zn concentration is high in seminal plasma and exceeds the blood mean values 30 times (23). Significant differences in seminal Zn concentrations have been detected between fertile and infertile men. This metal is a co-factor of Cu, Zn-SOD and it was supposed that the reduction in Zn concentration disrupted antioxidant defence, making the spermatozoa more susceptible to oxidative damage of membrane lipids, proteins, and DNA (24). Therefore the positive relationship between increased seminal Zn content and sperm production, motility, and morphology is reasonable. In fact, in the molecule of Cu, Zn-SOD, Zn^2+^ has a structural, stabilizing role, while in the catalytic activity Cu^2+^ is directly involved. SOD is one of the most important antioxidative defence enzymes. It catalyses the conversion of superoxide radicals to H_2_O_2_, which in turn could be degraded by GPx or catalase to the harmless oxygen molecule and water. This mechanism prevents the lipid peroxidation of the plasma membrane. If it is impossible to eliminate H_2_O_2_ from the cell, it can interact with a trace metal ion and generate the highly reactive OH radicals. Therefore the action of SOD should be conjugated with that of catalase or GPx to prevent the H_2_O_2_ action (25).

In regard to copper concentration, a number of animal and human studies found an inverse association between high Cu levels and semen quality (17,26). On the other hand, the concentration of Cu could influence the activity of copper-containing enzymes such as SOD. Abdul Rasheed (27) suggested a direct relationship between the level of copper in seminal plasma and the activity of SOD. In azoospermic patients he observed a significant decrease in the levels of seminal plasma copper that may lead to a concomitant decrease in SOD activity. Thus the decreased copper content may be considered an important mechanism of increased oxidative stress in azoospermic semen. Therefore maintenance of the microelements balance is crucial because both deficiency and excess may have negative consequences.

The studied elements (Fe, Cu, Zn, and Se) belong to the group of microminerals. Along with vitamins, they are regarded as the main class of micronutrients in the diet. Several studies have reported an improvement of sperm quality and increase of pregnancy rates when men changed the diet or took certain vitamins and micronutrients (28). Therefore a better comprehension of the trace elements’ mechanism of action and their effects on male fertility would contribute to the medication of male infertility. The levels of trace elements in the normozoospermic group as well as in various categories of abnormal sperm should be well identified, because both a deficiency and excess of these elements can disrupt the biological equilibrium and impact on fertility. Respectively an imbalance on one side or another could be restored with appropriate treatment. We detected a nearly significant correlation between age and Se (r=-0.545, p=0.067), as well as between Zn and morphology (r=-0.513, p=0.088), that required a further study with a larger number of people.

In conclusion, our results established alterations in the concentration of iron, zinc, and selenium in abnormal groups compared to normozoospermic group. These trace elements act as co-factors of the antioxidant enzymes CAT, SOD, and GPx, respectively, and their availability could affect the oxidative status of sperm. In addition, iron could act as a prooxidant via Fenton reaction. The impaired oxidative balance of spermatozoa could contribute to poor sperm motility and morphology, since in all abnormal groups an increased lipid peroxidation and decreased tGSH levels were detected. We found a significant correlation between the Fe levels and the tested markers of oxidative stress. Further research on the relationship between antioxidant enzyme activities and the level of their trace elemental co-factors in semen as well as their impact on the sperm parameters is required. Improved knowledge about the role of trace metals in male infertility could be useful in overcoming this serious problem in our contemporary life.

## Figures and Tables

**Table 1:**
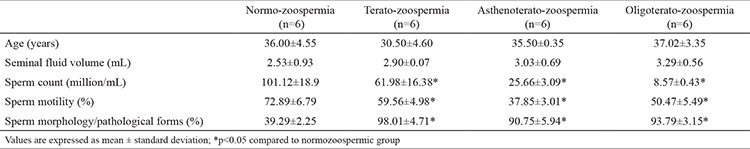
Patients’ age and sperm parameters in the groups

**Table 2:**
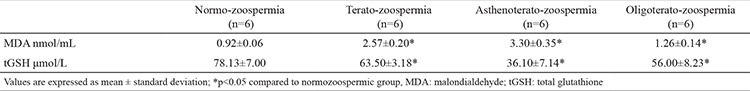
Concentration of malondialdehyde and total glutathione in seminal plasma in the groups

**Table 3:**
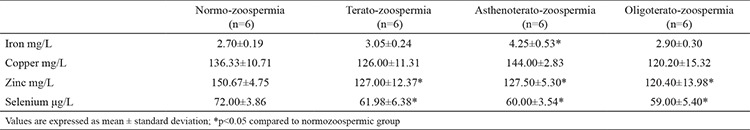
Concentration of iron, copper, zinc, and selenium in seminal plasma in the groups
